# Supplementary material for: Fine-tuning Large Language Models in Behavioral Psychology for Scalable Physical Activity Coaching
Source: medRxiv. 2025 Feb 21:2025.02.19.25322559. Preprint. [Version 1] doi: 10.1101/2025.02.19.25322559 (PMC11875315; doi:10.1101/2025.02.19.25322559)
Supplement: 1 [file NIHPP2025.02.19.25322559V1-supplement-1.pdf]

medRxiv preprint doi: https://doi.org/10.1101/2025.02.19.25322559; this version posted February 21, 2025. The copyright holder for this preprint (which was not certified by peer review) is the author/funder, who has granted medRxiv a license to display the preprint in perpetuity.
It is made available under a CC-BY 4.0 International license .

Supplemental Materials

**Stage of Change**	**Fine-Tuned LLM Health Coach Message**	**Human Expert Message**
Precontemplation	Get moving, feel alive! Short daily walks can reduce stress and increase energy, no fitness goals required, just start!	Try out something new today and surprise yourself with some physical activity. Who knows, maybe you will actually enjoy it?
Contemplation	Unleash your inner athlete! Exercise can help you discover new passions and interests. Try something new and have fun with it.	If others can do it, so can you. Try out a sport you haven't done before and figure out what you like the most.
Preparation	Get ready to sweat! Create a workout schedule, and let's get moving, your 30-day fitness journey starts now, are you ready?	Try to set some personal goals. Keep in mind that they have to be realistic and reachable.
Action	Get accountability and support! Share your fitness goals with a friend and schedule 30-minute walks together, 3 times a week.	It's often more fun to exercise together. Ask your friends to join you.
Maintenance	Remember why you started? Exercise daily to feel more energized, focused, and in control of your life.	If you continue now, feeling comfortable with your own body is going to be a steady aspect of your normal life.
Generic	Kick-start your day with a 10-min walk to reach 10,000 steps! Take a short break at work, walk to a coworker's desk, or do a few laps around your house.	The longer you push exercise aside, the longer you are denying yourself a better quality of life.

Supplemental Table 1: Examples of messages generated by the fine-tuned LLM Health Coach and human experts, tailored to different stages of behavior change. Expert messages were gathered from a crowdsourcing study involving 25 specialists in fitness, behavior, and health psychology.
